# Design and Rationale of Routine **U**ltrasou**N**d Gu**I**dance for **V**ascular Acc**E**ss fo**R** Cardiac Procedure**s**: **A** Randomized Tria**L** (UNIVERSAL)

**DOI:** 10.1016/j.cjco.2022.08.011

**Published:** 2022-08-30

**Authors:** Sulaiman Alrashidi, Marc-André d’Entremont, Omar Alansari, Jose Winter, Bradley Brochu, Laura Heenan, Elizabeth Skuriat, Jessica Tyrwhitt, Michael Raco, Michael B. Tsang, Nicholas Valettas, James Velianou, Tej Sheth, Matthew Sibbald, Shamir R. Mehta, Natalia Pinilla-Echeverri, Jon David Schwalm, Madhu K. Natarajan, Andrew Kelly, Elie Akl, Sarah Tawadros, Mercedes Camargo, Walaa Faidi, Gustavo Dutra, Sanjit S. Jolly

**Affiliations:** a McMaster University, Hamilton, Ontario, Canada; b Hamilton Health Sciences, Hamilton, Ontario, Canada; c Niagara Health, St. Catharines, Ontario, Canada; d Population Health Research Institute, Hamilton, Ontario, Canada; e Centre Hospitalier Universitaire de Sherbrooke (CHUS), Sherbrooke, Quebec, Canada; f Clinica Alemana de Santiago, Santiago, Chile; g CK Hui Heart Centre, Royal Alexandra Hospital, Edmonton, Alberta, Canada; h McGill University Health Centre, Montreal, Quebec, Canada

## Abstract

**Background:**

A significant limitation of femoral artery access for cardiac interventions is the increased risk of vascular complications and bleeding compared to radial access. Ultrasound (US)-guided femoral access may reduce major vascular complications and bleeding. We aim to determine whether routinely using US guidance for femoral arterial access for coronary angiography or intervention will reduce **B**leeding **A**cademic **R**esearch **C**onsortium (BARC) 2, 3, or 5 bleeding or major vascular complications.

**Methods:**

The **U**ltrasou**n**d Gu**i**dance for **V**ascular Acc**e**ss fo**r** Cardiac Procedure**s**: **A** Randomized Tria**l** (UNIVERSAL) is a multicentre, prospective, open-label, randomized trial with blinded outcomes assessment. Patients undergoing coronary angiography with or without intervention via a femoral approach with fluoroscopic guidance will be randomized 1:1 to US-guided femoral access, compared to no US. The primary outcome is the composite of major bleeding based on the BARC 2, 3, or 5 criteria or major vascular complications within 30 days. The trial is designed to have 80% power and a 2-sided alpha level of 5% to detect a 50% relative risk reduction for the primary outcome based on a control event rate of 14%.

**Results:**

We completed enrollment on April 29, 2022, with 621 randomized patients. The patients had a mean age of 71 years (25.4% female), with a high rate of comorbidities, as follows: 45% had a prior percutaneous coronary intervention; 57% had previous coronary artery bypass surgery; and 18% had peripheral vascular disease.

**Conclusions:**

The UNIVERSAL trial will be one of the largest randomized trials of US-guided femoral access and has the potential to change guidelines and increase US uptake for coronary procedures worldwide.

## Résumé

**Introduction:**

Par rapport à l’abord radial, la limitation importante de l’abord artériel fémoral lors des interventions au cœur pose un risque accru de complications vasculaires et de saignements. L’abord fémoral guidé par ultrasons (US) peut contribuer à réduire les complications vasculaires majeures et les saignements. Nous avons pour objectif de déterminer si l’utilisation systématique du guidage par US pour l’abord artériel fémoral lors des angiographies ou des interventions coronariennes contribuera à réduire les saignements de type 2, 3 ou 5 selon le ***B****leeding****A****cademic****R****esearch****C****onsortium* (BARC) ou les complications vasculaires majeures.

**Méthodes:**

*L’****U****ltrasou****n****d Gu****i****dance for****V****ascular Acc****e****ss fo****r****Cardiac Procedure****s****:****A****Randomized Tria****l*** (UNIVERSAL) est un essai multicentrique, prospectif, ouvert, à répartition aléatoire, réalisé par une évaluation à l’insu des résultats. Les patients subissant une angiographie coronarienne avec ou sans intervention par voie fémorale sous guidage fluoroscopique seront répartis de façon aléatoire 1:1 à l’abord fémoral guidé par US ou sans US. Le principal critère d’évaluation est le critère composite de saignements majeurs de type 2, 3 ou 5 selon les critères du BARC ou de complications vasculaires majeures dans les 30 jours. L’essai est conçu de façon à avoir une puissance de 80 % et un seuil alpha bilatéral de 5 % pour déterminer la réduction du risque relatif de 50 % du critère d’évaluation principal selon un taux d’événements dans le groupe témoin de 14 %.

**Résultats:**

Le 29 avril 2022, nous avons terminé le recrutement de 621 patients choisis aléatoirement. Les patients avaient un âge moyen de 71 ans (25,4 % de femmes) et un taux élevé de comorbidités : 45 % avaient déjà subi une intervention coronarienne percutanée, 57 % avaient déjà subi un pontage aorto-coronarien et 18 % avaient une maladie vasculaire périphérique.

**Conclusions:**

L’essai UNIVERSAL qui sera l’un des plus vastes essais à répartition aléatoire sur l’abord fémoral guidé par US a le potentiel de faire changer les lignes directrices et de faire augmenter le recours aux US lors des interventions coronariennes dans le monde entier.

Although radial access reduces major bleeding events and vascular complications, compared to femoral access, in patients requiring coronary interventions, femoral access is still needed for large-bore procedures or when the radial arteries are too small or occluded.^1^^,^^2^ As a result, strategies to improve the safety of femoral access are needed.

Cannulation above the inguinal ligament is associated with an increase in retroperitoneal hemorrhage, whereas cannulation below the common femoral artery (CFA) bifurcation is associated with an increased risk of arteriovenous fistula formation and pseudoaneurysm.^3^, ^4^, ^5^ The use of ultrasound (US)-guided access showed promise in earlier trials, including the **F**emoral **A**rterial Access with **U**ltra**s**ound **T**rial (FAUST). However, the more recent **S**tandard vs **U**ltrasound-Guided **R**adial and **F**emoral Access in Coronary Angiography and Intervention (SURF) trial showed no reduction in vascular complications or bleeding with US guidance.^6^

Two surveys of interventional cardiologists demonstrated that only 13%-27% of operators routinely used US guidance for femoral access, despite 88% answering that US was available in the catheterization laboratory.^7^^,^^8^ Improving the safety of femoral access is needed, especially as the patients receiving femoral access are a small subset of complex patients for whom radial access is not chosen due to technical reasons. These patients often have the highest bleeding risk.

**U**ltrasou**n**d Gu**i**dance for **V**ascular Acc**e**ss fo**r** Cardiac Procedure**s**: **A** Randomized Tria**l** (UNIVERSAL) was designed as a large simple trial to compare US guidance with no US guidance on a background of fluoroscopic guidance for femoral arterial access for coronary angiography or intervention.

## Primary Objective

The primary objective of the study is to determine whether the use of a US-guided approach compared to no US for femoral arterial access for coronary procedures on a background of fluoroscopic guidance will reduce the rate of the composite outcome of major bleeding, based on the **B**leeding **A**cademic **R**esearch **C**onsortium (BARC) 2, 3, or 5 criteria, or major vascular complications within 30 days.

## Hypothesis

Although fluoroscopy readily identifies the location of the femoral head, US allows the operator to visualize the bifurcation of the common femoral artery into superficial and profunda femoral arteries, theoretically reducing the risk of cannulating above the inguinal ligament and below the bifurcation. Furthermore, US may permit the operator to avoid puncturing areas of heavy calcium and disease, potentially reducing complications during cannulation and with closure devices. Using US may reduce the number of punctures and reduce the risk of puncture of branch vessels or venous structures, thereby potentially reducing bleeding and vascular complications.

## Design

UNIVERSAL is a multicentre, parallel randomized open-label trial with blinded outcome assessment comparing US-guided access to no US guidance in patients undergoing coronary angiography or percutaneous coronary intervention (PCI) on a background of fluoroscopic guidance in both groups. The trial is coordinated by the Population Health Research Institute at Hamilton Health Sciences, McMaster University, Hamilton, Ontario, Canada, and has been approved by local ethics boards. The trial is registered at ClinicalTrials.gov with identifier number NCT03537118. UNIVERSAL is an investigator-initiated trial, and funding was provided by the McMaster University Division of Cardiology Innovation Fund and the Hamilton Health Sciences Foundation.

## Eligibility Criteria

Patients were eligible if they were referred for coronary angiography or PCI with planned femoral access. The only exclusion criteria were being aged ≤ 18 years, having had ST-elevation myocardial infarction as the initial presentation, and the absence of a palpable femoral pulse (Table 1).

**Table 1:** Eligibility criteria

Inclusion criteria
1. Patients referred for coronary angiography with planned upfront femoral access
2. Elective and non-ST-segment elevation acute coronary syndrome patients
Exclusion criteria
1. Age ≤ 18 years
2. Absence of palpable femoral pulse
3. ST-elevation myocardial infarction patients

## Randomization

Using a centralized computer randomization service (ROME, Population Health Research Institute, Hamilton, Ontario, Canada), we randomized patients 1:1, stratified by planned closure-device use, to femoral arterial access either with US vs with no US. All patients were to have fluoroscopy of the femoral head prior to puncture, in both groups. We considered patients randomized as soon as the treatment allocation was provided to the operator. Allocation was blinded with a variable block size.

## Operator Requirements

Operator expertise is important for US-guided access.^9^ Operators were required to have completed at least 10 US-guided femoral access procedures and to demonstrate that they had achieved all the steps listed below to demonstrate proficiency.

## Treatment Groups

The treatment groups were as follows: (i) experimental group: US-guided femoral access; (ii) control group: no US.

### US

After locating the femoral head with fluoroscopy, the operator used a sterile covered transducer (5 to 10 MHz linear array) and followed the sequence outlined below.

(i) Locate and identify the CFA bifurcation into the superficial and profunda femoral arteries and the femoral head.
(ii) Scan for atherosclerosis and identify a site free of anterior calcification and plaque suitable for access.
(iii) Perform local anesthesia with xylocaine.
(iv) Follow the 18-gauge needle as it enters the artery in the short-axis plane.
(v) Confirm the position with wire in the artery before sheath insertion in orthogonal views: long axis over the femoral head and short axis with the wire in the centre of a disease-free cross-section above the bifurcation.
(vi) Once the operator is satisfied with the entry site, insert the femoral introducer.

### Control group

After locating the femoral head with fluoroscopy, the operator obtained access through palpation of the femoral artery prior to local anesthetic injection.

## Procedures

For both treatment groups, an observer recorded the number of attempts to access the femoral artery, the time between local anesthesia and insertion of the femoral sheath, and the presence of venipunctures. The location of the introducer was evaluated with a peripheral angiogram in the left anterior oblique (LAO) or right anterior oblique (RAO) projections at the level of the femoral head to assess the relation of the femoral introducer with the femoral head and femoral bifurcation. Micropuncture was not mandated in the trial because the prior randomized **Fe**moral **M**icropuncture **o**r **R**outine **I**ntroducer **S**tudy (FEMORIS, N = 402) did not show a reduction in access-site bleeding.^10^ As a result, the use of micropuncture was left up to the operator and was recorded on case report forms.

## Study Outcomes

### Primary outcome

The primary outcome is the composite of BARC 2, 3, or 5 bleeding or major vascular complications (femoral artery pseudoaneurysm, arteriovenous fistula, retroperitoneal bleed, large hematoma of more than 5 cm in diameter, or ischemic limb requiring intervention or surgery) within 30 days (Table 2).^11^

**Table 2:** Primary composite outcome definitions

Term	Definition
**Major vascular complication**	
Femoral artery pseudoaneurysm	Diagnosis must be confirmed by US performed by a radiologist demonstrating the following characteristics^27^: 1. Two-dimensional US shows an echo lucent sac that expands and contracts with the cardiac cycle; 2. Color Doppler demonstrates a swirling flow pattern with turbulence inside the echo lucent sac; 3. Pulsed wave Doppler displays a “to-and-fro” signal.
Arteriovenous fistula	Diagnosis must be performed by a radiologist using contrast computed tomography or Doppler US confirming blood flow from the femoral artery into the femoral vein.
Retroperitoneal bleed	Diagnosis must be performed by a radiologist confirming the presence of a hematoma in the retroperitoneal space.
Large hematoma	Diagnosis must be performed by a clinician or nurse, confirming a diameter ≥ 5 cm.
Ischemic limb	Ischemic limb is defined as limb-threatening ischemia, which is confirmed by limb hemodynamics or imaging, and leads to an acute vascular intervention (ie, pharmacologic [heparin or thrombolysis], peripheral artery surgery, peripheral artery angioplasty, or amputation) within 30 days of the procedure.
**Major bleeding**	
BARC 2 bleeding^11^	Any overt, actionable sign of hemorrhage that does not fit the criteria for type 3, 4, or 5 but does meet at least one of the following criteria: 1. Requiring intervention by healthcare personnel; 2. Leading to hospitalization or medical consultation; 3. Requiring medical evaluation.
BARC 3 bleeding^11^	Type 3a 1. Over bleeding plus a hemoglobin drop of 3 to < 50 g/L 2. Overt bleeding requiring transfusion Type 3b 1. Overt bleeding plus a hemoglobin drop of ≥ 50 g/L 2. Cardiac tamponade 3. Bleeding requiring surgical intervention for control 4. Bleeding requiring intravenous vasoactive agents Type 3c 1. Intracranial hemorrhage 2. Intraocular bleed compromising vision
BARC 5 bleeding^11^	Type 5a 1. Probable fatal bleeding Type 5b 1. Definite fatal bleeding with overt bleeding, imaging confirmation, or autopsy

BARC, Bleeding Academic Research Consortium; CT, computed tomography; US, ultrasound.

### Other outcomes

The key secondary outcomes include the individual components of the primary outcome. Other outcomes include the number of attempts to achieve arterial puncture, successful CFA cannulation, venipunctures, closure device failure, and the total time to obtain femoral access.

## Study Procedures

At discharge, a blinded investigator assessed the presence of a major vascular complication or major bleeding. We also perform a comprehensive chart review and telephone follow-up within 30 days postprocedure, to assess for complications.

### Angiographic core laboratory evaluation

Blinded research personnel will analyze peripheral angiograms in an angiographic core laboratory located at the Hamilton General Hospital, Hamilton, Ontario, Canada. We will tabulate the location of the femoral bifurcation, the presence of femoral artery calcification, the site of femoral artery cannulation, active bleeding, and femoral artery dissection.

## Statistical Considerations

The initial sample size was based on the definition used in the **R**ad**i**al **v**s Femor**al** Access for Coronary Angiography or Intervention (RIVAL) trial in patients with acute coronary syndromes of major vascular complications, and the study used a 3% expected event rate in the control arm and a 1% expected event rate in the experimental arm. These assumptions led to a sample size of 1538 patients, in order to have 80% power with a 2-sided 5% type 1 error level.^12^

A limitation of our initial sample size calculation is that BARC 2 bleeding information was not collected in the RIVAL trial, and therefore it did not have an accurate assessment of event rates. As a result, we re-estimated our sample size based on blinded event rates after enrolling 450 patients. Our revised control event rate was estimated to be 14%. Subsequently, 600 patients were needed to have 80% power, a 2-sided 5% type 1 error level, and a 50% relative risk reduction for the primary outcome. We amended the protocol on April 12, 2021. Power calculations were performed using PASS (version 13, NCSS Statistical Software, Kaysville, UT). Recruitment took longer than expected due to the COVID-19 pandemic halting all non-COVID-19-related research during periods of 2020.

For the primary analysis, an intention-to-treat analysis will include all randomized patients.^13^ In this way, patients will be kept in the treatment group to which they were originally randomized. A secondary as-treated analysis will also be performed. A χ^2^ test or Fisher’s exact test will be performed on a per-patient basis, and statistical significance will be claimed if the *P* value is less than 0.05. Of note, core-lab analyses will be performed on a per-access basis. Odds ratios and 95% confidence intervals will also be reported to quantify treatment effects.

## Subgroup Analysis

We will perform multiple a priori subgroup analyses to identify potential differing treatment effects in the following groups: age ( ≥ 75 years vs < 75 years), sex (male vs female), body mass index ( ≥ 30 kg/m^2^ vs < 30 kg/m^2^), peripheral vascular disease (presence vs absence), clinical presentation (non-ST elevation acute coronary syndrome vs elective), coronary intervention (PCI vs angiography alone), type of procedure (chronic total occlusion procedure vs nonchronic total occlusion procedure), sheath size ( ≥ 7 French vs < 7 French), planned closure device (planned vs not planned), and actual closure device use (used vs not used). The underlying hypothesis is that US-guided access will be more beneficial to higher-risk groups. No statistical adjustment will be made for multiple testing.

## Baseline Characteristics

The baseline characteristics of the 621 randomized patients are shown in Table 3. The patients had a mean age of 71 years; 25.4% were female; and they had a mean body mass index of 30.3 kg/m^2^. The trial population had a high rate of comorbidities: 51% had a prior myocardial infarction; 42% had diabetes; 45% had prior PCI; 57% had previous coronary artery bypass surgery; 19% had atrial fibrillation; and 18% had peripheral vascular disease. Chronic total occlusion PCI was performed in 14% of patients.

**Table 3:** Baseline characteristics for **U**ltrasou**n**d Gu**i**dance for **V**ascular Acc**e**ss fo**r** Cardiac Procedure**s**: **A** Randomized Tria**l** (UNIVERSAL) participants

Variables	Overall
Randomized, n	621
**Demographics and comorbidities**	
Age, y, mean (SD)	70.56 (10.24)
Female sex	158 (25.4)
BMI, kg/m^2^, mean (SD)	30.31 (23.26)
Hypertension	538 (86.6)
Dyslipidemia	555 (89.4)
Diabetes	261 (42.0)
Current smoker	92 (14.8)
Previous myocardial infarction	319 (51.4)
Previous PCI	278 (44.8)
Previous CABG	353 (56.8)
Previous stroke/TIA	58 (9.3)
Peripheral vascular disease	110 (17.7)
Atrial fibrillation	115 (18.5)
Chronic kidney disease	93 (15.0)
PCI performed during procedure	262 (42.2)
**Indication for procedure**	
Atypical chest pain	83 (13.4)
Stable angina	156 (25.1)
Silent ischemia	17 (2.7)
Unstable angina	55 (8.9)
NSTEMI	123 (19.8)
Planned PCI	32 (5.2)
CTO PCI	88 (14.2)
Valvular assessment	12 (1.9)
Other	87 (14.0)
**Indication for femoral access**	
Absent radial pulse	78 (12.6)
Small radial artery	32 (5.2)
Previous CABG	306 (49.3)
Patient preference	8 (1.3)
Physician preference	64 (10.3)
Large-bore procedure	15 (2.4)
CTO PCI	103 (16.6)
Prior failed radial access	3 (0.5)
Other	37 (6.0)
**Medications at baseline**	
Aspirin	520 (83.7)
Plavix	250 (40.3)
Ticagrelor	70 (11.3)
Prasugrel	0 (0.0)
Warfarin	24 (3.9)
NOACs	78 (12.6)
Fondaparinux	39 (6.3)
Unfractionated heparin	22 (3.5)
Low-molecular-weight heparin	8 (1.3)
Bivalirudin	1 (0.2)
GPIIb/IIIa inhibitors	0 (0.0)

Values are n (%), unless otherwise indicated.

BMI, body mass index; CABG, coronary artery bypass surgery; CTO, chronic total occlusion; GPIIb/IIIa, glycoprotein IIb/IIIa; NOAC, novel oral anticoagulant; NSTEMI, non-ST-segment elevation myocardial infarction; PCI, percutaneous coronary intervention; SD, standard deviation; TIA, transient ischemic attack.

## Discussion

The UNIVERSAL trial will determine the effect of routine US guidance on a background of fluoroscopic guidance for femoral access for coronary angiography and intervention on BARC 2, 3, or 5 bleeding and major vascular complications. Although US-guided access is ubiquitous in interventional radiology, interventional cardiology surveys show that it is used in 13%-27% of coronary procedures, suggesting that further data are needed to change practice.^7^^,^^8^

A few randomized trials have examined the use of US guidance for femoral access.^14^, ^15^, ^16^, ^17^ The FAUST trial randomized 1004 patients to fluoroscopic vs US-guided access and demonstrated no difference in the primary outcome of CFA cannulation rates (86.4% vs 83.3%, *P* = 0.17).^15^ However, the first-pass success rate increased and the number of attempts was reduced, leading to a decrease in vascular complications favoring US access (1.4% vs 3.4%, *P* = 0.04), primarily driven by hematomas. However, a subsequent meta-analysis did not demonstrate a significant difference in major bleeding outcomes (0.3% vs 1.3%; odds ratio, 0.28; 95% confidence interval, 0.07-0.1.16; *P* = 0.08) or minor bleeding (1.4% vs 2.8%; odds ratio, 0.50; 95% confidence interval, 0.24-1.05; *P* = 0.07).^18^

The SURF trial, a 2 x 2 factorial randomized trial of 1388 patients (radial vs femoral and standard vs US), showed a benefit for radial vs femoral access, but no difference for US guidance for the composite endpoint of major bleeding, major cardiovascular events, or vascular complications at 30 days.^6^ The trial did show significant procedural efficacy differences favouring US access. In the femoral subgroup, US-guided access vs no US-guided access did not significantly reduce vascular complications or major bleeding. A meta-analysis including this trial showed no reduction in major bleeding but did show a reduction in vascular complications.^19^

The large UNIVERSAL trial will inform clinical decision-making regarding femoral access for coronary interventions.^15^ Although it is similar to the FAUST trial, key differences include the following: use of clinical outcome as the primary outcome instead of CFA cannulation; use of a blinded angiographic core laboratory for robust and unbiased peripheral angiogram analysis; and utilization of modern BARC bleeding definitions with blinded assessment. Radial is the dominant access in the participating centres in the UNIVERSAL trial. As a result, the included patients were complex, elderly, high-risk patients for whom radial access was not used due to patient or procedural characteristics, thereby increasing the risk profile of patients compared to that in prior trials. Furthermore, based on the blinded interim event rates, the UNIVERSAL trial has significantly higher numbers of events than the FAUST trial and thus may have greater power to detect clinically important differences in clinical outcomes.

The current American College of Cardiology/American Heart Association/Society for Cardiovascular Angiography and Interventions (SCAI), and European Society of Cardiology guidelines do not specifically mention recommendations regarding technical aspects for achieving femoral access, including US guidance.^20^^,^^21^ However, the SCAI recommends that US guidance be considered to decrease bleeding complications.^22^ Furthermore, several other evidence-based reviews recommend US guidance, and the SCAI has excellent advice on preventing and managing femoral access complications.^23^, ^24^, ^25^, ^26^ The results of the UNIVERSAL trial may provide further evidence needed to develop more definitive recommendations in upcoming clinical guidelines (ie, Class 1 recommendation) and help change practice.

### Limitations

The 50% reduction in the primary outcome was derived from meta-analyses; however, the UNIVERSAL trial is not powered for more modest but clinically important differences. A larger trial powered for smaller effect sizes would have been optimal but was not possible due to limitations in funding. Finally, US only addresses the method of entry; how closure is performed is important and is another avenue for preventing bleeding complications.

### Study status

We completed enrollment on April 29, 2022, with 621 randomized patients. Data collection and statistical analyses are ongoing, with complete data expected to be presented in September 2022.

## Conclusion

The UNIVERSAL trial is a large, simple trial designed to assess the effect of routine US-guidance during femoral access for coronary angiography or interventions on BARC 2, 3, or 5 bleeding or major vascular complications.
